# Improvement of Mechanical Properties of Rubberized Cement-Stabilized Macadam by Optimization of Rubber Particle Gradation

**DOI:** 10.3390/ma18225106

**Published:** 2025-11-10

**Authors:** Donghai Wang, Shuxing Mao, Chaochao Liu, Jie Chen

**Affiliations:** 1 National Engineering Research Center of Highway Maintenance Technology, Changsha University of Science & Technology, Changsha 410114, China; sryx1299@126.com (D.W.); maoshuxing0730@stu.csust.edu.cn (S.M.); jc@stu.csust.edu.cn (J.C.); 2 Hunan Provincial Architectural Design Institute Group Co., Ltd., Changsha 410000, China; 3 Guangxi Transportation Science and Technology Group Co., Ltd., Nanning 530007, China

**Keywords:** rubberized cement-stabilized macadam, rubber particle gradation, toughness enhancement, fatigue properties, crack resistance

## Abstract

Replacing natural aggregates in cement-stabilized macadam (CSM) with waste rubber particles reduces mineral resource consumption, manages solid waste, and enhances the long-term performance of cementitious materials, addressing environmental challenges. An optimized gradation of rubber particles was proposed based on different combinations of particle sizes. Five rubber particle combinations with different gradations were incorporated into CSM to create a rubberized cement-stabilized macadam (RCSM). The strength of RCSM was verified through compressive and flexural tensile tests. The toughness of RCSM was evaluated using the flexural ultimate failure strain and flexural tensile resilient modulus. Crack resistance was evaluated through freeze–thaw, fatigue, and shrinkage tests. The results indicate that the compressive and flexural strengths of RCSM with 1.18–4.75 mm rubber particles are closest to those of CSM. The ultimate strain of CSM increased by up to 1.83 times with optimized rubber gradation, while its modulus decreased by more than half. Furthermore, RCSM with 1.18–4.75 mm rubber particles exhibited the best performance in fatigue life under high stress ratio, frost resistance, and shrinkage behavior. Comprehensive test results showed that rubber particles ranging from 1.18 to 2.36 mm were most effective in improving the road performance of RCSM.

## 1. Introduction

Accumulating scrap rubber tires is a significant global environmental problem [1,2,3]. Around 1 billion waste tires are generated annually worldwide, with estimates suggesting this could rise to 1.2 billion by 2030 [4,5,6]. Due to rubber’s non-biodegradable nature, improper disposal can negatively impact ecological and environmental health [7]. In recent decades, recycled rubber particles have been used as modifiers in asphalt binders and as substitutes for natural aggregates in concrete production [8,9,10]. Studies show that wet-processed crumb rubber modified asphalt mixtures offer improved cracking, rutting, and fatigue resistance [11,12,13]. However, it should be noted that the addition of crumb rubber substantially increases the viscosity of asphalt binders [14], which complicates the mixing and placement processes. Therefore, there exists a practical upper limit on the crumb rubber content, beyond which the workability and technological performance of the binder deteriorate. Additionally, incorporating rubber into concrete has been shown to enhance toughness, durability, and functional properties, including improved impact resistance, freeze–thaw performance, and ductility [15,16,17,18,19,20,21,22,23,24,25]. Nonetheless, other challenges may occur, including reduced compressive strength, delayed setting, and weakened interfacial bonding between rubber particles and the cementitious matrix, all of which merit further investigation.

Reusing discarded rubber addresses disposal issues and conserves natural aggregates, benefiting the environment and the economy [26]. Cement-Stabilized Macadam (CSM) is the main material used for China’s semi-rigid base of high-grade highway pavements. Because CSM is brittle and highly sensitive to temperature and moisture variations, it cannot effectively dissipate thermal and drying shrinkage stresses, frequently leading to shrinkage cracks [27,28]. Additionally, its limited toughness leads to deformation incompatibility with the overlying asphalt layers. This increases the concentration of tensile stresses at the bottom of the semi-rigid subgrade, ultimately causing fatigue cracking under repeated loads. Therefore, developing high-toughness semi-rigid base materials is crucial for improving crack resistance and ensuring long-lasting asphalt pavements.

Methods for improving the toughness of cement-based materials mainly include fiber reinforcement and rubber modification. Fiber-reinforced concrete (FRC) has recently seen widespread use, such as polypropylene fiber concrete, basalt fiber concrete, alkali-resistant glass fiber concrete, and steel fiber concrete [29,30]. However, FRC is still not fully effective in controlling crack propagation. While FRC offers greater toughness than traditional concrete, it still exhibits strain-softening behavior under direct tensile loading, often leading to wider and more damaging cracks [31,32]. Engineered cementitious composites (ECC) [33] are concrete materials with lower fiber content, demonstrating strain-hardening behavior, such as PE-ECC [34] and PVA-ECC [35]. Similarly, ultra-high-toughness cementitious composites (UHTCC) [36] further enhance crack control and exhibit superior strain-hardening performance compared to FRC. It was found that [37] about 0.3% volume fraction of PVA fibers can increase concrete’s compressive and splitting tensile strength by 15.75% and 36.09%, respectively, compared to plain concrete.

Scrap rubber tires, classified as environmental pollutants, exhibit good elasticity and a low stiffness modulus. Incorporating rubber particles into cement-based materials significantly enhances toughness, offering performance and environmental benefits. This indicates that rubber particles significantly reduce the stiffness of concrete. Additionally, Goulias [38] found that the brittle damage of rubber concrete specimens improved as the rubber content increased. Hou [39] et al. reported that smaller and more uniformly graded rubber particles can more effectively control crack width. The same principle applies to Rubber-Cement-Stabilized Macadam (RCSM). Ahmed [40] reported that the flexural strength and stiffness of RCSM decreased after adding rubber. Under splitting and bending loads, the ultimate strains of RCSM are 1.9 and 3.79 times greater than those of conventional CSM, respectively. Despite the reduction in strength, the significant increase in toughness effectively improves crack resistance in a semi-rigid base.

However, weak adhesion between rubber particles and the cement matrix creates numerous defects in the interfacial transition zone (ITZ), likely accelerating the failure of the hardened mixture. The hydrophobic nature of rubber particles is the main cause of their weak bond with hardened cement paste. Surface modification has been shown to enhance the bond between rubber and cement. Research shows that washing with water can remove impurities from the rubber surface [36]. Pre-coating rubber surfaces with cementitious materials forms hydrophilic cement shells, improving the bond between rubber aggregates and the cement matrix [41].

The bond strength is strongly influenced by particle size, with smaller rubber particles generally forming stronger interfaces. Yu et al. [42] demonstrated that the strength of concrete mixed with 2–4 mm crumb rubber decreased less than that of concrete mixed with 60–80 mesh (0.18–0.28 mm) powdered rubber. Piti [43] reported that the shrinkage rate of rubber concrete using large-sized rubber particles is typically higher than that of concrete using small-sized rubber particles. Yu et al. [44] stated that 1–3 mm rubber particles were more effective at inhibiting cracking than those with sizes of 2–4 mm and less than 2 mm. Although particle size strongly influences the behavior of rubberized cement-based materials, its effect has not been systematically investigated. Most studies have focused on rubber concrete, with limited research in CSM.

This research aims to explore the correlation between rubber particle size and the road performance of RCSM. The goal is to develop RCSM with enhanced toughness and superior crack resistance through optimal rubber gradation. Different rubber particle size combinations were designed and incorporated into RCSM. Unconfined compressive strength, four-point bending, freeze–thaw, and shrinkage tests were conducted on RCSM. The impact of rubber particle size distribution on the strength and toughness of RCSM was evaluated through laboratory experiments. Meanwhile, the optimal rubber particle size range for RCSM was proposed. This paper proposes a method that significantly enhances the deformability and durability of CSM while maintaining strength. This method is significant for efficiently using waste rubber and for environmental protection.

## 2. Materials

### 2.1. Cement

The cement used in the experiment was Ordinary Portland cement (P.O.32.5R) (Huaxin Cement Co., Ltd., Yueyang, China). Table 1 provides details of its properties and test results.

### 2.2. Aggregates

**Figure 1:**
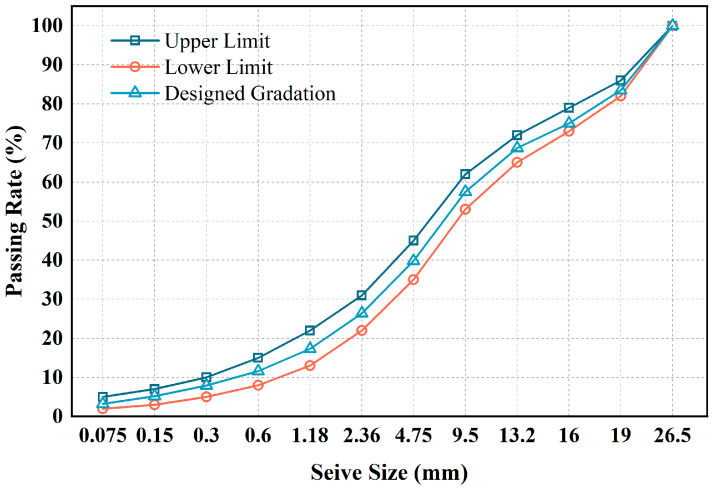
Aggregate gradation curve.

In this study, CSM was prepared using basalt aggregates. Table 2 and Table 3 present the basic parameters of coarse and fine aggregates, which comply with the requirements of Chinese standards JTG E42-2005 [45]. In addition, the gradation of mineral aggregates is depicted in Figure 1.

### 2.3. Rubber Particles

**Figure 2:**
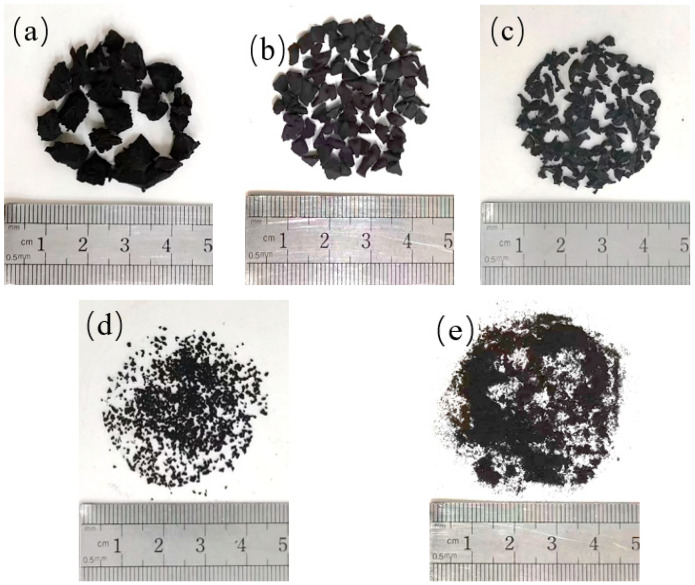
Rubber particles: (**a**) R1: 4.75–9.5 mm (**b**) R2: 2.365–4.75 mm (**c**) R3: 1.185–2.36 mm (**d**) R4: 0.65–1.18 mm (**e**) R5: 0.155–0.3 mm.

As shown in Figure 2, the five different sizes of rubber particles are labeled R1, R2, R3, R4, and R5. The R1-R5 rubber particle sizes were 4.75–9.5 mm, 2.36–4.75 mm, 1.18–2.36 mm, 0.6–1.18 mm, and 0.15–0.3 mm, respectively. The performance parameters are listed in Table 4.

## 3. Mix Proportion Design and Specimen Preparation

### 3.1. Mix Proportion Design of RCSM

Given the low specific gravity of rubber particles, the mixture volume may expand significantly if the aggregates are replaced with the same mass of rubber. This volume expansion can cause mutual interference among aggregates and disrupt the original gradation composition. Therefore, RCSM was prepared by substituting rubber particles for aggregates of the same size through isovolumetric replacement, based on the mineral aggregate gradation shown in Figure 1. The replacement method involves calculating the mass of rubber to be added through Equation (1) and determining the mass of aggregates to be replaced through Equation (2).

(1) $$\frac{M_{R}}{M}=X$$

(2) $$\frac{\rho_{A}}{\rho_{R}}M_{R}=M_{A}$$

where *X* is the rubber dosage (%); $\rho_{R}$ and $\rho_{A}$ refer to the densities of rubber and aggregate, respectively (g/cm^−3^); $M_{R}$ represents the mass of rubber (g); M is the total mass of the original aggregates (g); $M_{A}$ stands for the mass of aggregates replaced (g).

In this study, the rubber dosage was fixed at 3% by volume to balance the strength and toughness requirements of cement-stabilized macadam (CSM). Preliminary trials and references [46] showed that rubber contents exceeding 5% led to a sharp reduction in compressive strength, falling below the JTG E42-2005 specification limit. Conversely, contents below 2% yielded negligible improvement in toughness. Therefore, a 3% replacement was adopted as an optimal value to achieve both structural integrity and enhanced deformability. The densities $\rho_{R}$ and $\rho_{A}$ were 1.10 g/cm^3^ and 2.67 g/cm^3^. Based on Equation (3), the aggregate substitution ratio was calculated as 7.3 vol%, corresponding to an equal-volume replacement between aggregates and rubber particles. Specifically, replacing 7.3 vol% of the same-sized aggregate with 3 wt% rubber (based on the total aggregate mass) ensures the same overall mixture volume. However, the proportions of aggregates with different particle sizes in the mineral aggregate varied considerably. According to Figure 1, the detailed proportions of different aggregate size fractions are listed in Table 5.

(3) $$\frac{M_{A}}{M}=\frac{\rho_{A}}{\rho_{R}}X$$

**Figure 3:**
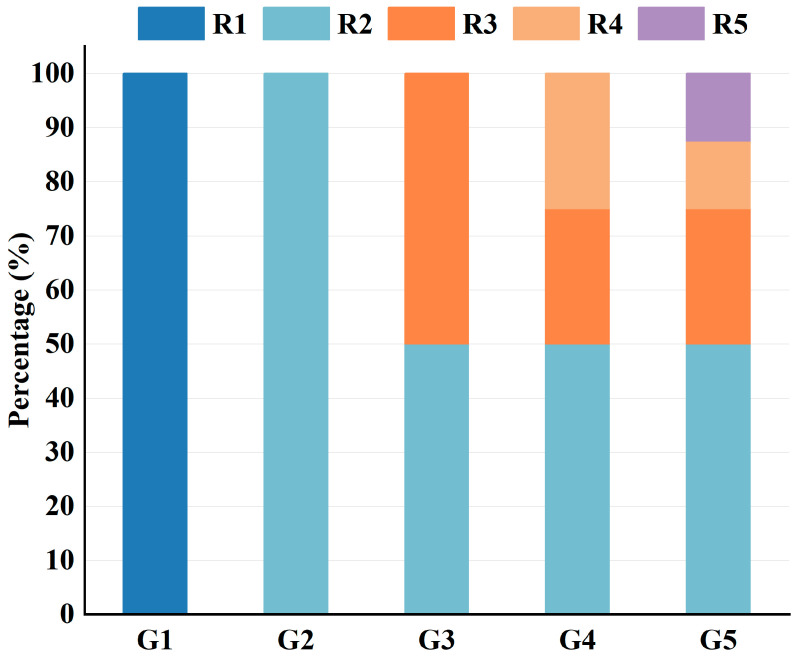
Rubber particle gradation composition.

**Figure 4:**
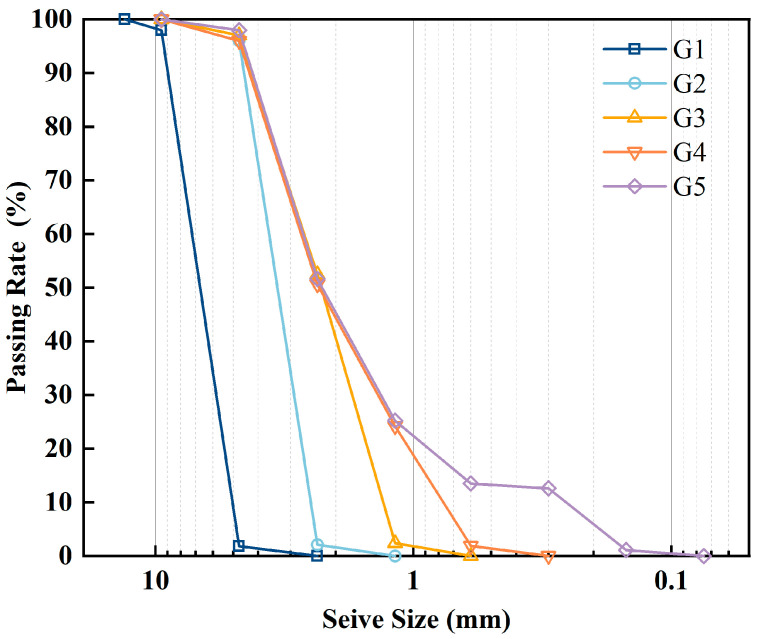
Rubber particle gradation curve.

As shown in Table 5, 41 vol% of the 4.75–9.5 mm aggregates in the original gradation were replaced by R1 rubber particles (this value was obtained by multiplying the proportion of the 4.75–9.5 mm aggregate in the total gradation, 17.7%, by the calculated aggregate substitution ratio, 7.3 vol%). However, the replacement ratio of aggregate replaced by R3 was up to 80 vol%. For the 0.6–1.18 mm aggregate, due to their low proportion in the gradation, the replacement ratio exceeds 100%. If the rubber particle’s gradation remains unchanged, the aggregates are insufficient for replacement. Therefore, a rubber gradation design method was proposed in this study to address this issue. The percentage of rubber with different sizes in the five gradations (named G1 to G5) was designed as shown in Figure 3. The mix ratio was designed to maintain the total amount of rubber particles at 3% of the total aggregate mass. The 4.75–9.5 mm and 2.36–4.75 mm rubber gradation schemes were consolidated into a new particle size. Based on the single particle size rubber grading scheme of 2.36–4.75 mm, finer rubber particles (1.18–2.36 mm) were introduced at a content of 1.5%, creating a new gradation as show in Figure 4. Since then, the content has been set to half the previous rubber content for each incorporation of a smaller rubber particle. This method replaced finer aggregates, combining rubber particles of various sizes.

The mix proportions of RCSM with various rubber gradations and CSM were determined using a vibration compaction test. The cement content for all mixtures was 4.5%. The results for the optimum moisture content and maximum dry density are presented in Table 6. 

### 3.2. Specimen Preparation

Based on the maximum dry density results, the masses of constituents in regular CSM were determined and are presented in Table 7. The masses of components in RCSM with five different rubber gradations were determined.

**Figure 5:**
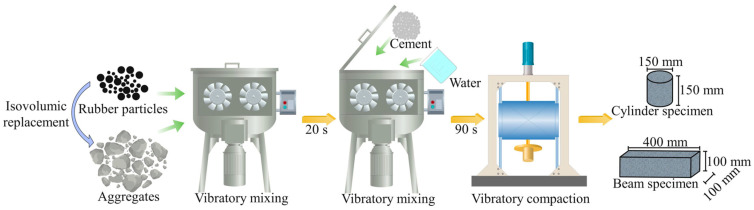
Specimen preparing process of RCSM.

Due to the significant density difference between rubber and mineral aggregates, rubber particles tend to float to the surface of the mixture during mixing. Prepared by the vibration mixing method, rubber particles can be uniformly dispersed within the RCSM specimens. Therefore, all specimens were prepared using the vibration mixing method. The vibration compactor was set to a frequency of 30 Hz and an excitation force of 7.6 kN. The unconfined compressive strength tests for the mixture specimens before and after 7 days, 28 days, and freeze–thaw cycles were conducted using cylindrical specimens with a height and diameter of 150 mm each. Bending strength tests, bending modulus of resilience tests, fatigue tests, dry shrinkage tests, and temperature shrinkage tests were performed using beam specimens. Each specimen measures 400 mm in length, 100 mm in width, and 100 mm in height. The compaction time for cylindrical specimens was 90 s, while beam specimens required 120 s of vibration. Subsequently, the specimens were transferred to the standard curing room. The detailed preparation process is shown in Figure 5.

### 3.3. Experimental Procedure

#### 3.3.1. Unconfined Compressive Strength Test

Compressive strengths of six mixture types—including five RCSM with different rubber gradations and one CSM control group—were tested by ASTM C109. The specimens were cured under standard conditions (temperature: 20 ± 2 °C, humidity: ≥95%) for 7 and 28 days. Four cylindrical specimens (ϕ150 mm × 150 mm) were tested using the Landmark electrohydraulic servo testing system from MTS Systems Corporation of the United States (YAW-2000B, Located in Changsha, China) for each mixture type. Loading was uniformly and continuously applied at a 1 mm/min rate. Four replicate specimens were included in each group.

#### 3.3.2. Flexural Tensile Strength Test

In accordance with standard JTG E60-2008 [47], the flexural tensile strength and ultimate flexural failure strain of RCSM specimens were evaluated through a four-point bending test using the Material Testing System (MTS). Four-point bending tests were conducted on beam specimens (400 mm × 100 mm × 100 mm) cured for 90 days. The MTS loading rate was controlled at 50 mm/min, spanning between supports of 357 mm. The test results for each set of samples were determined by averaging the values measured from four specimens.

#### 3.3.3. Flexural Tensile Resilient Modulus Test

The flexural tensile resilient modulus of mixed specimens cured for 90 days was measured using four-point bending tests. In accordance with the standard JTG E60-2008, a dynamic load with a Haversine continuous semi-positive vector waveform was applied at a frequency of 10 Hz. A five-stage loading protocol was set, with peak loads reaching 0.1P, 0.2P, 0.3P, 0.4P, and 0.5P, respectively (where P is the ultimate flexural-tensile failure load). The load was applied gradually from low to high, with 200 cycles at each level, without breaks. The loading rate was controlled at 1 mm/min, and six parallel specimens were utilized.

#### 3.3.4. Fatigue Test

The fatigue loading was performed using the MTS machine. The dynamic periodic continuous semi-positive vector load was utilized. The loading frequency *f* was 10 Hz, with a period *T* of 0.1 s. The fatigue test was conducted at room temperature. Four stress levels of 0.75 MPa, 1 MPa, 1.25 MPa, and 1.5 MPa were examined. The test specimens were beam specimens measuring 400 mm × 100 mm × 100 mm, cured for 90 days. Three groups of parallel specimens were prepared for each test level.

The relationships between fatigue life and stress ratio or stress level were established based on the S-N fatigue models (Equations (4) and (5)).

(4) $$\mathrm{lg}N_{f}=\mathrm{lg}k-n\mathrm{lg}\sigma$$

(5) $$\mathrm{lg}N_{f}=\mathrm{lg}k-n\mathrm{lg}t$$

where $N_{f}$ is the fatigue life; σ is the stress level; *t* is the nominal stress ratio, i.e., the ratio of the stress level to the standard strength; *k* and *n* are the parameters of the fatigue prognostic model.

#### 3.3.5. Freeze–Thaw Test

The average strength values of four specimens were calculated before and after freeze–thaw cycles to evaluate frost durability. After being cured for 28 days, the cylindrical specimens were frozen at −18 °C for 16 h and then thawed in water at 20 °C for 8 h. After five freeze–thaw cycles, the unconfined compressive strength was tested using YAW-2000B.

#### 3.3.6. Temperature Shrinkage Test

The performance of six mixture types in resisting temperature cracks was evaluated by conducting a temperature shrinkage test. After curing for 7 days, the beam specimens were placed in a drying oven at 120 °C for 12 h. Afterward, the specimens were transferred to a well-ventilated environment until they reached room temperature. Before testing, the specimen was placed on the shrinkage meter, and the micrometer was set to zero. The specimens and shrinkage instruments were both placed in a temperature-alternating chamber. The temperature gradually decreased from 60 °C at a cooling rate of 0.5 °C/min, and for every 10 °C drop, it was maintained for 3 h. This procedure was repeated until the temperature dropped to −20 °C. The micrometer readings were recorded during the last five minutes of each 3 h, and four parallel specimens were used for each mixture.

#### 3.3.7. Drying Shrinkage Test

The water-saturated beam specimens were placed on the shrinkage instrument after 7 days of curing. The experimental temperature was maintained between 20 °C and 25 °C. During the first 7 days of the experiment, the dial indicator readings were recorded daily. Subsequently, changes in specimen length were measured every two days from day 8 to day 30, and the quality of the specimen was tested. After the test, the specimens were dried in an oven until they reached a constant weight. The six parallel specimens’ shrinkage deformation and water loss rate were recorded during each testing cycle.

Each test was repeated three times, and the average values are reported herein. The standard deviations are shown as error bars in the Figures. The coefficients of variation for strength and strain results were within 5%, indicating good repeatability and reliability of the experimental data.

## 4. Results and Discussion

### 4.1. Unconfined Compressive Strength

**Figure 6:**
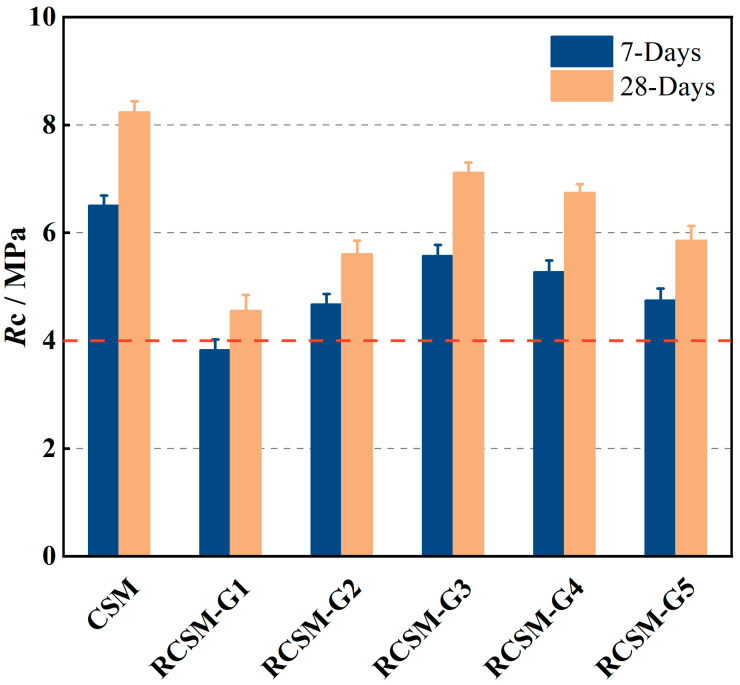
Unconfined compressive strength of mixtures.

The compressive strength results of RCSM at various curing ages are illustrated in Figure 6. Compared to CSM, the incorporation of rubber particles significantly reduced the compressive strength, attributed to their poor strength and hydrophobic nature. With the original tougher aggregates replaced by rubber particles, the strength of RCSM would definitely decrease. Furthermore, the hydrophobic rubber particles failed to form strong interfacial bonds with the cement paste, weakening the overall strength.

For RCSM-G1, the compressive strength losses at 7 days and 28 days were 41% and 45% compared to CSM, respectively, significantly higher than other RCSM. Moreover, the 7-day compressive strength of RCSM-G1 was below 4 MPa, failing to meet specification requirements. One possible reason for the significant strength deterioration is the absence of coarse aggregates in RCSM-G1. Coarse aggregates, typically larger than 4.75 mm, form a strong skeletal structure when embedded and locked together within the mixture. The strength of RCSM relies heavily on this skeletal structure, but replacing coarse aggregates with rubber particles substantially weakens it. Additionally, another possible reason is that coarse rubber particles negatively impact the compaction performance of the mixture. During compaction, rubber absorbs energy and undergoes resilient deformation, resulting in poor compaction. As a result, the strength of RSCM-G1 was significantly compromised, suggesting that RCSM should not be manufactured with rubber particles larger than 4.75 mm.

From RCSM-G1 to RCSM-G5, the compressive strength exhibited a hump-shaped trend, with the inflection point occurring at RCSM-G3. The 7-day and 28-day compressive strength of RCSM-G3 remained above 85% of regular CSM, indicating that the use of 1.18–4.75 mm rubber particles provided excellent strength retention. Furthermore, the strength retention of 1.18–2.36 mm rubber particles was superior to that of 2.36–4.75 mm particles, as demonstrated by the comparison between RCSM-G2 and RCSM-G3.

According to RCSM-G4 and RCSM-G5, the strength of the mixture began to decrease when rubber particles smaller than 1.18 mm were added. The primary reason may be the reduction in the content of silty aggregates in the mixture. When water infiltrates, silty aggregates tend to bond with surrounding aggregates due to capillary pressure. Consequently, capillary pressure was reduced when the silty aggregates were partially replaced by hydrophobic rubber particles, which ultimately decreased strength.

The interfacial behavior between rubber particles and CSM aggregates can be further explained from a hydrophilic–hydrophobic adhesion perspective. Rubber is hydrophobic and non-polar, whereas the cementitious matrix is hydrophilic and dominated by polar hydration products. This mismatch weakens chemical bonding at the rubber–mortar interface, resulting in lower strength but allowing greater deformation and crack-dissipation capacity.

### 4.2. Flexural Tensile Test

#### 4.2.1. Flexural Tensile Strength

**Figure 7:**
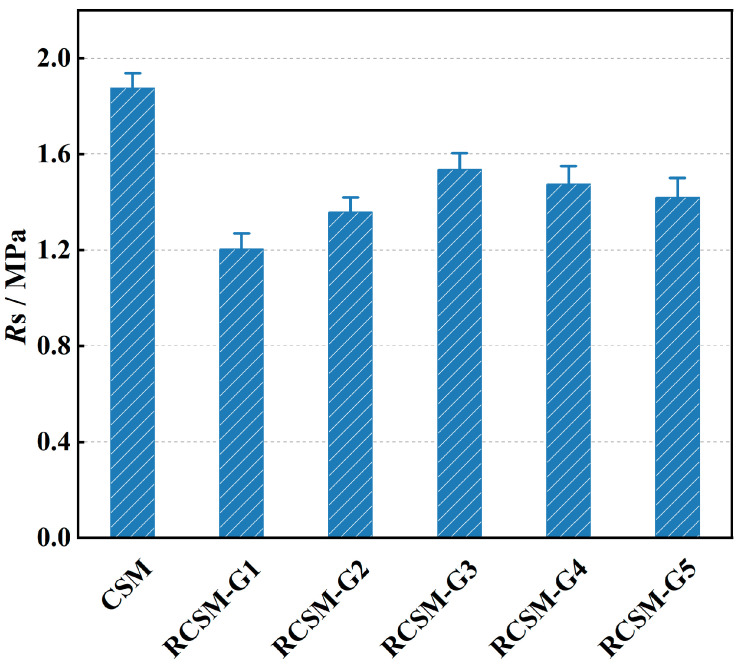
Flexural tensile strength of mixtures.

As shown in Figure 7, incorporating rubber particles reduced the flexural tensile strength of CSM by 18–36%, mainly due to the weak interfacial bonding between the hydrophobic rubber and the cement matrix. The reduction was most pronounced in RCSM-G1, whose strength was only about 64% of that of CSM. This can be attributed to the loss of the coarse aggregate skeleton structure and the formation of voids within the interfacial transition zone caused by large rubber particles (>4.75 mm).

Among the tested mixtures, RCSM-G3 exhibited the highest flexural strength retention, reaching approximately 82% of CSM. Its strength was 9% higher than that of RCSM-G2, indicating that rubber particles in the range of 1.18–4.75 mm provide an optimal balance between strength and flexibility. When finer particles (<1.18 mm) were used (RCSM-G4 and RCSM-G5), flexural strength decreased further, as excessive fine rubber weakened the aggregate bonding and increased internal porosity.

#### 4.2.2. Flexural Ultimate Failure Strain

**Figure 8:**
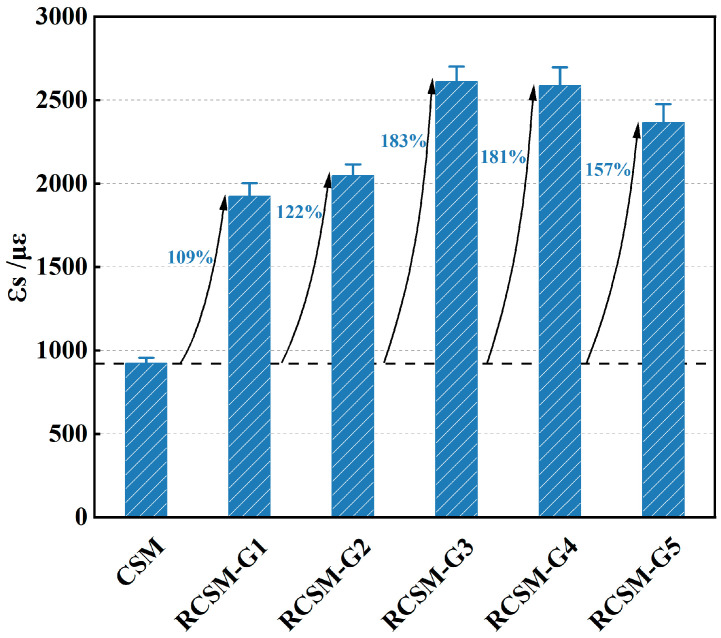
Flexural ultimate failure strain of mixtures.

As illustrated in Figure 8, incorporating rubber particles enhanced the deformation capacity and toughness of RCSM. The flexural ultimate failure strain exhibited a hump-shaped pattern across RCSM-G1 to RCSM-G5. RCSM-G1 demonstrated the smallest increase in ultimate strain, approximately 2.1 times that of CSM. Rubber particles larger than 4.75 mm could not embed into the aggregate skeleton during compaction, so they continuously absorbed stress and exhibited resilient deformation. This impeded the adequate compaction of the mixture. Therefore, replacing coarse aggregate with large rubber particles is not a viable approach for improving the toughness of RCSM.

The ultimate failure strain of RCSM-G3 was nearly 2.8 times that of regular CSM, indicating the highest toughness improvement. The gradation of RCSM-G3 consisted of 1.18–4.75 mm rubber particles, effectively filling the voids in the aggregate skeleton under vibratory loading. After compaction, the mixture achieved good contact, forming numerous interfacial contact points between rubber particles and aggregates. Consequently, stress within the mixture was efficiently transferred to and absorbed by the rubber particles.

According to test results from RCSM-G4 and RCSM-G5, the ultimate strain of RCSM decreased when rubber particles smaller than 1.18 mm were incorporated. Based on this result, the lower limit of rubber particle size for toughness improvement was determined to be 1.18 mm.

### 4.3. Flexural Tensile Resilient Modulus

**Figure 9:**
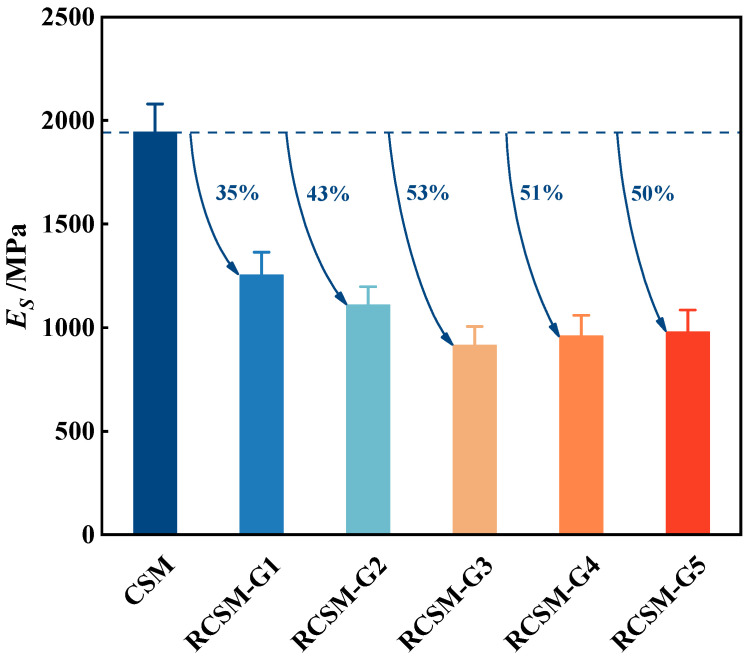
Flexural tensile resilient moduli of mixtures.

Figure 9 indicates that RCSM exhibits greater flexibility compared to CSM. Given that the stiffness modulus of tire rubber ranges from 0.47 to 2.7 MPa [48], the modulus of the multi-phase material varies depending on the rubber composition and volume fraction. Therefore, the modulus of RCSM can be adjusted by controlling the rubber gradation. The moduli of RCSM-G1 and RCSM-G2 were approximately 64% and 57% of that of CSM, respectively, with a smaller reduction than RCSM-G3. The results suggest that the range of RCSM modulus adjustment with a single rubber particle size is considerably narrower than that of mixed rubber gradations. The modulus of CSM can be reduced by altering the gradation of rubber particles. Furthermore, rubber particles between 1.18 and 2.36 mm were found to be the most effective in relieving the stiffness of CSM among all the tested rubber particles.

### 4.4. Fatigue Properties

Table 8 shows that for the same mixture, the *n* values derived from the fatigue equation based on stress ratio and stress level are quite similar, and the *n* values across different mixtures show slight variation. However, the *k* values differ significantly among the mixtures. The *k* value of the CSM fatigue curve is lower than that of the other RCSM fatigue curves, indicating that, under the same nominal stress ratio, RCSM has a longer fatigue life than CSM. Data from Table 9 reveal that the *k* value of the CSM fatigue curve associated with stress levels is relatively high, implying that CSM can withstand repeated loading as effectively as RCSM under the same stress conditions.

Additionally, whether based on stress ratio or stress level, the k value of the fatigue curve for the mixture with combined particle size rubber gradation is significantly higher than that of the single particle size gradation. Specifically, the RCSM-G3 mixture exhibits the highest *k* value among all tested mixtures, demonstrating that optimizing rubber particle gradation not only enhances the strength and toughness of CSM but also significantly improves its fatigue performance.

### 4.5. Freeze–Thaw Test

**Figure 10:**
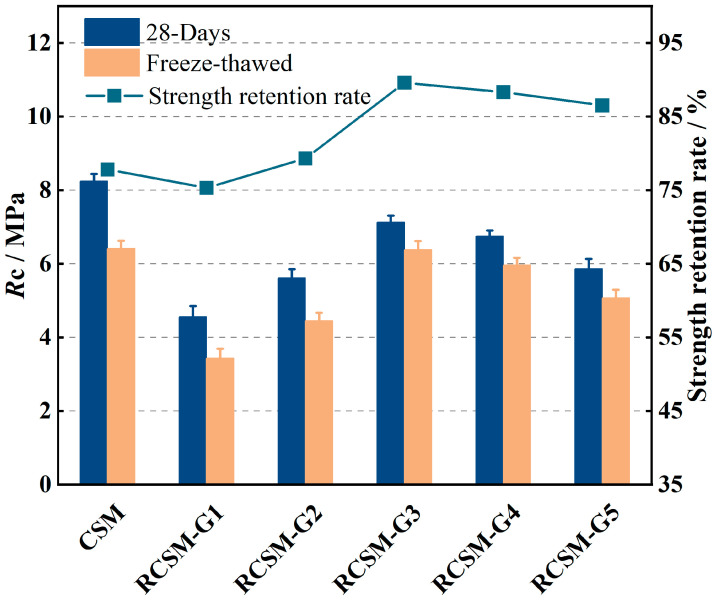
Freeze–thaw test results of different RCSM.

The freeze–thaw test results are provided in Figure 10. After five freeze–thaw cycles, the unconfined compressive strength of the mixtures decreased significantly. Meanwhile, the strength retention rate (the ratio of strength after to before freeze–thaw) indicates that, except for RCSM-G1, the strength retention of RCSM is superior to that of CSM. RCSM-G1 experienced the most severe frost swelling due to the large voids within the mixture.

The strength of RCSM-G3 decreased from 7.12 MPa before freeze–thaw to 6.38 MPa afterward, resulting in a strength retention of approximately 89.6%. This demonstrates that rubber particles with sizes between 1.18 and 4.75 mm effectively enhanced the freeze–thaw durability of RCSM. Water freezing caused volume expansion, generating expansion stress within the mixture during the freeze–thaw process. Nonetheless, elastic rubber particles could prevent this stress from acting directly on the CSM through stress absorption. Additionally, in RCSM-G4 and RCSM-G5, incorporating rubber particles smaller than 1.18 mm deteriorated the frost resistance durability. The wide range of rubber particle sizes caused excessive seepage pressure within the mixture. Water in the capillaries contained soluble salts, which created a concentration gradient between pores of different sizes. As the free water temperature in the pores drops to the freezing point, unfrozen water is continuously adsorbed and accumulates due to seepage pressure. Consequently, significant seepage pressure was generated within the ITZ.

### 4.6. Temperature Shrinkage

**Figure 11:**
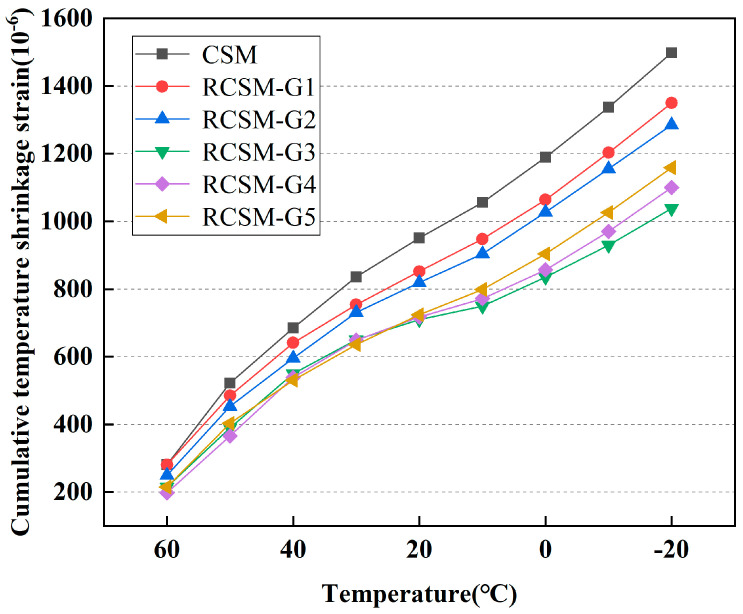
Temperature dependence of temperature shrinkage strain.

As shown in Figure 11, the cumulative temperature shrinkage strain increased as the temperature rose. Owing to their excellent deformability, rubber particles can effectively absorb the temperature-induced shrinkage stress in CSM. The cumulative strain of RCSM was notably lower than that of CSM, indicating that adding rubber particles reduced the temperature-induced shrinkage deformation in semi-rigid subgrade materials. Consequently, the anti-cracking performance of the semi-rigid subgrade was markedly improved.

CSM exhibited the highest cumulative strain of 1217 με among all the mixtures tested. The cumulative strain of RCSM decreased with the addition of rubber particles. Rubber particles absorb the shrinkage stresses caused by temperature decreases, thus reducing the temperature shrinkage strain of mixtures. Specifically, the cumulative strain of RCSM-G1 and RCSM-G2 decreased by 20.3% and 15.4%, respectively, compared to CSM. RCSM-G1 and RCSM-G2 contain larger rubber particles, which are less well-dispersed than fine particles in the mixtures. Therefore, the mitigation effect of single-sized rubber particles on temperature shrinkage in the mixture is less effective than that of rubber particles with combined gradations. Furthermore, no additional improvement in temperature-induced shrinkage strain was observed in RCSM when rubber particles smaller than 1.18 mm were added. These findings suggest that the optimal rubber particle size for enhancing the temperature shrinkage performance of RCSM ranges from 1.18 mm to 2.36 mm.

### 4.7. Dry Shrinkage

**Figure 12:**
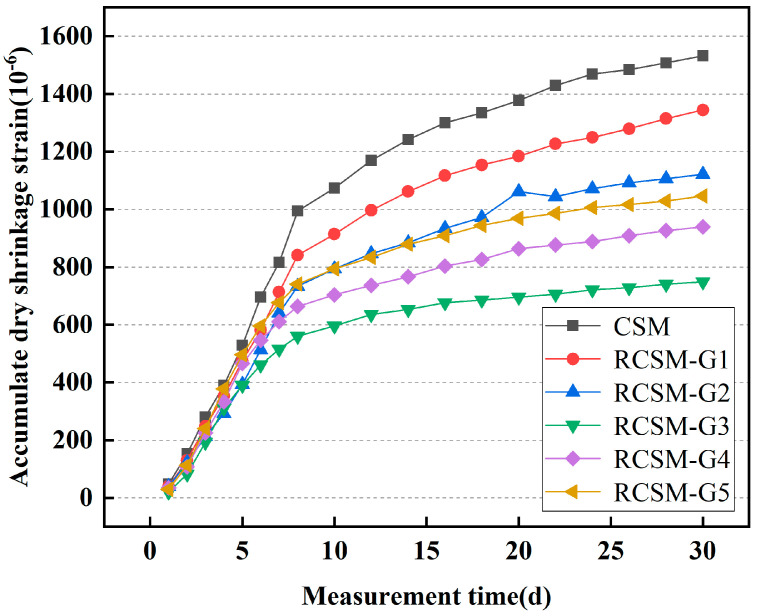
Cumulative dry shrinkage strain over time.

Figure 12 illustrates the cumulative dry shrinkage strain variation in RCSM over time. Generally, the drying shrinkage of base course materials primarily occurs during the early stages of paving. Therefore, the monitoring interval for this test was set between 1 and 30 days. The rate of increase in dry shrinkage strain for specimens was relatively high during the early stages, irrespective of whether rubber particles were added. After 7 days, the rate of increase began to slow, attributed to the significant loss of surface moisture during the initial stage of the test. Subsequently, the internal moisture starts to act due to adsorbed water and intermolecular forces. As a result, the water loss rate slows, and the cumulative dry shrinkage strain growth rate decreases.

After 30 days of continuous drying, the cumulative dry shrinkage strain of CSM reached 1532.3 με. By contrast, the cumulative dry shrinkage strains of RCSM-G1 to RCSM-G5 were 1344.5 με, 1121.7 με, 748.6 με, 938.9 με, and 1046.6 με, respectively. The cumulative dry shrinkage strain of RCSM-G3 was reduced by 51% compared to CSM, indicating that the rubber particles effectively reduced the dry shrinkage strain in CSM.

When the rubber gradation includes two or more particle sizes, the cumulative dry shrinkage strain of RCSM is lower than that of mixtures with single-sized rubber particles. This is primarily due to the excessive replacement of single particle sizes in RCSM-G1 and RCSM-G2, which significantly weakened the skeletal structure of CSM. However, including overly fine rubber particles increases the number of micro-pores in the mixture, resulting in more significant drying shrinkage stresses. Consequently, the cumulative dry shrinkage strain for RCSM-G4 and RCSM-G5 increased compared to RCSM-G3.

Table 10 summarizes the key results from Section 4. Overall, incorporating rubber particles reduced compressive and flexural strengths due to weaker interfacial bonding but significantly enhanced toughness, flexibility, and durability. The RCSM-G3 mixture, containing rubber particles sized between 1.18 and 4.75 mm, achieved the best balance among strength, fatigue resistance, and shrinkage performance. The correlations indicate that moderate stiffness reduction improves crack resistance and freeze–thaw durability through enhanced energy dissipation and stress absorption.

## 5. Conclusions

This study proposed an optimization method for rubber gradation. It addressed the inconsistency in gradation caused by unequal replacement particle sizes between rubber and aggregate. By optimizing rubber use in cement-based materials, the method enhances rubber utilization while improving the toughness and durability of cementitious materials. The main conclusions can be summarized as follows:

(1) Due to the weak bonding of the incorporated rubber particles, the flexural tensile strength and unconfined compressive strength of RCSM significantly decreased. Rubber particles larger than 4.75 mm or finer than 1.18 mm are not recommended to replace aggregates.

(2) The deformability and toughness of RCSM improved significantly with the introduction of flexible rubber particles. The ultimate failure strain of RCSM-G3 increased by 1.83 times compared to CSM. Additionally, rubber gradation optimization can reduce the flexural tensile modulus of RCSM to less than half that of CSM.

(3) Except for RCSM-G1, optimizing the rubber gradation significantly enhanced frost resistance durability and decreased the fatigue stress sensitivity of RCSM. The optimal rubber particle size for improving the road performance of RCSM ranged from 1.18 to 2.36 mm.

(4) The incorporation of rubber particles significantly reduced both the drying shrinkage strain and temperature shrinkage strain of RCSM. Moreover, RCSM with two or more rubber particle sizes exhibited a lower cumulative shrinkage strain than RCSM with single-sized particles.

(5) The incorporation of waste rubber particles into CSM significantly improves its long-term durability, offering a sustainable solution for reducing natural aggregate consumption in road construction.

This study establishes a quantitative optimization approach for rubber gradation in RCSM, revealing that rubber particles between 1.18 and 2.36 mm offer the best balance of strength and flexibility. The fatigue life of RCSM-G3 increased by approximately 1.8 times compared with conventional CSM, while frost resistance and shrinkage performance were significantly improved. These findings provide a practical reference for the design of crack-resistant semi-rigid base materials in highway pavements, contributing to the recycling of waste rubber and reduction in natural aggregate consumption. Although this study systematically analyzed the mechanical and durability properties of RCSM with optimized rubber gradation, certain limitations remain. The experimental program was conducted under laboratory conditions, and field verification of the long-term performance of RCSM in actual pavement structures has not yet been performed.

## Figures and Tables

**Table 1:** Basic properties of ordinary Portland cement.

Parameters		Test Result	Unit
Fineness		5.9	%
Specific surface area		364	m^2^/kg
Normal consistency		25.6	%
Loss on ignition		1.29	%
Setting time	Initial	198	min
	Final	258	min
Compressive strength	3 d	22.0	MPa
	28 d	34.8	MPa
Flexural strength	3 d	3.8	MPa
	28 d	7.1	MPa

**Table 2:** Basic parameters of coarse aggregates.

Parameters	Technical Requirements	Sieve Size (mm)			Unit
		4.75–9.5	9.5–19	19–26.5	
Crushed stone value	≤26	-	20.5	18.4	%
Flat and elongated particle	≤22	11.9	9.5	8.8	%
Particle content less than 0.075 mm	≤2	0.8	0.7	0.5	%
Soft stone content	≤5	4.1	3.7	2.9	%

**Table 3:** Basic parameters of fine aggregates.

Parameters	Technical Requirements	Test Results	Unit
Organic matter	≤2	0.6	%
Sulfate content	≤0.25	0.17	%
Particle content less than 0.075 mm	≤17	9	-

**Table 4:** Chemical and physical properties of rubber particles.

Properties	Rubber Particles				
	R1	R2	R3	R4	R5
Sieve size/(mm)	9.5–4.75	4.75–2.36	2.36–1.18	1.18–0.6	0.3–0.15
Density/(g/cm^3^)	1.10~1.13				
Water content/%	<1.0				
Ash content/%	<10				
Acetone extract content/%	<15				
Metal content/%	<0.08				

**Table 5:** Aggregate replacement rates for different sizes.

Size of Aggregates (mm)	Proportion (%)	Aggregate Substitution Ratio (%)
4.75–9.5	17.7	7.3
2.36–4.75	13.4	
1.18–2.36	9.1	
0.6–1.18	5.7	
0.15–0.3	2.7	

**Table 6:** Compaction test results.

Type	Optimum Moisture/%	Maximum Dry Density/(g/cm^−3^)
CSM	6.0	2.413
RCSM-G1	5.6	2.279
RCSM-G2	5.4	2.298
RCSM-G3	5.3	2.314
RCSM-G4	5.2	2.335
RCSM-G5	5.1	2.358

**Table 7:** Mix proportion of CSM and RCSM mixtures (wt%).

Type	Cement	Water	Aggregates	Rubber Particles				
				R1	R2	R3	R4	R5
CSM	4.06	5.42	90.52	/	/	/	/	/
RCSM-G1	4.09	5.09	88.17	2.65	/	/	/	/
RCSM-G2	4.09	4.91	88.35	/	2.65	/	/	/
RCSM-G3	4.1	4.83	88.41	/	1.33	1.33	/	/
RCSM-G4	4.1	4.74	88.51	/	1.33	0.66	0.66	/
RCSM-G5	4.11	4.65	88.58	/	1.33	0.67	0.33	0.33

**Table 8:** Fitting parameters of mixture fatigue equation based on stress ratio.

Mixture	lg*k*	*k*	*n*	*R* ^2^
CSM	1.42	26	9.72	0.995
RCSM-G1	2.45	282	8.33	0.996
RCSM-G2	2.64	437	9.03	0.979
RCSM-G3	2.78	603	8.84	0.997
RCSM-G4	2.73	537	9.12	0.996
RCSM-G5	2.73	537	9.18	0.988

**Table 9:** Fitting parameters of mixture fatigue equation based on stress level.

Mixture	lg*k*	*k*	*n*	*R* ^2^
CSM	4.07	11,749	9.77	0.997
RCSM-G1	3.13	1349	8.43	0.997
RCSM-G2	3.85	7079	9.02	0.998
RCSM-G3	4.43	26,915	8.82	0.998
RCSM-G4	4.26	18,197	9.12	0.996
RCSM-G5	4.13	13,490	9.19	0.989

**Table 10:** Summary of performance results and correlations of RCSM mixtures.

Property	Trend Compared to CSM	Optimal Rubber Gradation
Unconfined compressive strength	Decreased strength	RCSM-G3
Flexural tensile strength	Decreased strength	RCSM-G3
Flexural tensile resilient modulus	Fracture strain increases	RCSM-G3, RCSM-G4
Flexural tensile resilient modulus	Reduction in flexural modulus	RCSM-G3
Fatigue properties	Increased fatigue life	RCSM-G3
Freeze–thaw test	Strength retention rate increased	RCSM-G3
Temperature shrinkage	Cumulative strain reduction	RCSM-G1
Dry shrinkage	Cumulative dry shrinkage strain reduction	RCSM-G3

## Data Availability

The original contributions presented in the study are included in the article, further inquiries can be directed to the corresponding author.
